# Infant mortality and its determinants in Uganda 2016: Using a geographically weighted regression approach

**DOI:** 10.1371/journal.pgph.0002669

**Published:** 2023-12-20

**Authors:** Janis E. Campbell, Jessica Beetch, Townsend Cooper, Jianquan Cheng

**Affiliations:** 1 Department of Biostatistics and Epidemiology, Hudson College of Public Health, University of Oklahoma Health Sciences Center, Oklahoma City, OK, United States of America; 2 Department of Natural Sciences, Manchester Metropolitan University, Chester, United Kingdom; 3 Key Laboratory of Environment Change and Resources Use in Beibu Gulf, Centre for Health Geographic Information and Education, Nanning Normal University, Nanning, PR China; PLOS: Public Library of Science, UNITED STATES

## Abstract

Infant mortality (IM) represents the overall health of a country or region as it relates to access to medicine, health care, and clean water in a population. IM remains understudied in many areas of Uganda, as many studies are from urban the capital (Kampala). The long-term goal of this research is the mitigation of IM and poor pregnancy outcomes in Uganda. Insights gained from geographic distribution of IM will allow adaptation of diagnosis, treatments, and interventions within the studied areas. Through using OLS and geographically weighted regression, this study has explored the significant factors and their heterogeneous and scaling effects in 2016 across Uganda. The empirical findings from this study include a significant association between IM and both being unmarried and preferring to speak Luganda when interviewed. Those unmarried may lack a social network to assist with income, childcare, and household chores representing decreased resources. Additionally, being interviewed in Luganda was associated over a large geographic area, which may represent not being comfortable in English, which is the language of education, commerce, and presumably health care, thus suggesting a disconnect with health care settings. These data suggest that strides can be made in Uganda by providing targeted resources to areas with high rates of unmarried mothers and those areas with high rates of Luganda as their language of choice.

## Introduction

Infant mortality (IM), or death of an infant under one year of age, represents the overall health of a country at one point in time and allows for comparisons of population health over time [1]. IM is an important factor in population health as factors that impact infant death, such as access to medicine, health care, and clean water, also affect the health of others in the population [1]. Most (98%) of the 2017 infant deaths in the world occurred in low and middle-income countries [2,3]. In 2017, estimates showed that Sub-Saharan Africa had the highest neonatal mortality rates (death under 28 days/1000 live births) in the world at 27.2 for every 1000 births overall [4]. In Uganda 2018 estimates ranged from 27,889 to 46,361 total infant deaths [5]. These disparities and their sequelae have been previously described in parts of Uganda and other resource-limited settings [6–12].

Uganda has made significant improvements in mitigating IM. The mortality rates in Uganda were, by far, much higher in 1990 as compared to the global average rate. According to a UNICEF report, 18.4% of the children born in Uganda in 1990 died. The proportion was reduced to 4.6% in 2018 [13]. To further improve Uganda’s IM mitigation requires introducing policies that would enhance the awareness and ability of parents to provide necessary care. Some factors, such as poverty and illiteracy, are social issues that influence more than IM rates. Understanding the risk factors for IM can enhance the formulation of strategies for future mitigation. For example, one of the Ugandan government’s actions to reduce the rate of child mortality included introducing the country-wide free prenatal and postnatal services [14].

Geographically weighted regression (GWR) allows for the development of local level estimates and compares them to their neighbors to produce maps and statistics that look at the processes behind spatial heterogeneity, while still comparing to a larger unit or the global process. A GWR model can be considered a type of regression model with geographically varying parameters. One of GWR’s strengths is that it can be used in estimating parameters anywhere within the study area provided a dependent variable along with a set of one or more variables is known. Another strength of the GWR is its applicability in examining the geographical patterns of long-term conditions, which is useful in limiting long-term poor health outcomes. In this case, the long-term condition includes those that might have stemmed from exposure to work-related risks, poor housing conditions, lack of social support (being unmarried), and stress-related conditions (unintended pregnancy, young maternal age). Application of GWR in the investigation of the relationships between infant deaths and social phenomenon quantitatively helps in investigating whether there is an existing stable relationship over space or whether they vary to reflect the features of different localities within the study area, possibly representing unmeasured variables that fluctuate geographically [15]. GWR allows stimulus-response visualization and an understanding of how the relationship changes in space.

IM remains understudied within Uganda, as many of the previously cited studies are from the capital (Kampala) or central Uganda [9,16]. While several studies have looked at entire country [6,14,17,18] or rural areas, [7,11,18] none used GWR to understand the differing patterns associated with IM. Through using GWR, the long-term objective of this project is to explore geographic patterns of IM in Uganda particularly focusing on unique geographic factors associated with IM. The aims of this study were to characterize the geographic distribution of IM and associated factors in 2016 Uganda, adjusting for infant, maternal, and community risk factors.

## Methods and materials

### Setting

**Fig 1:**
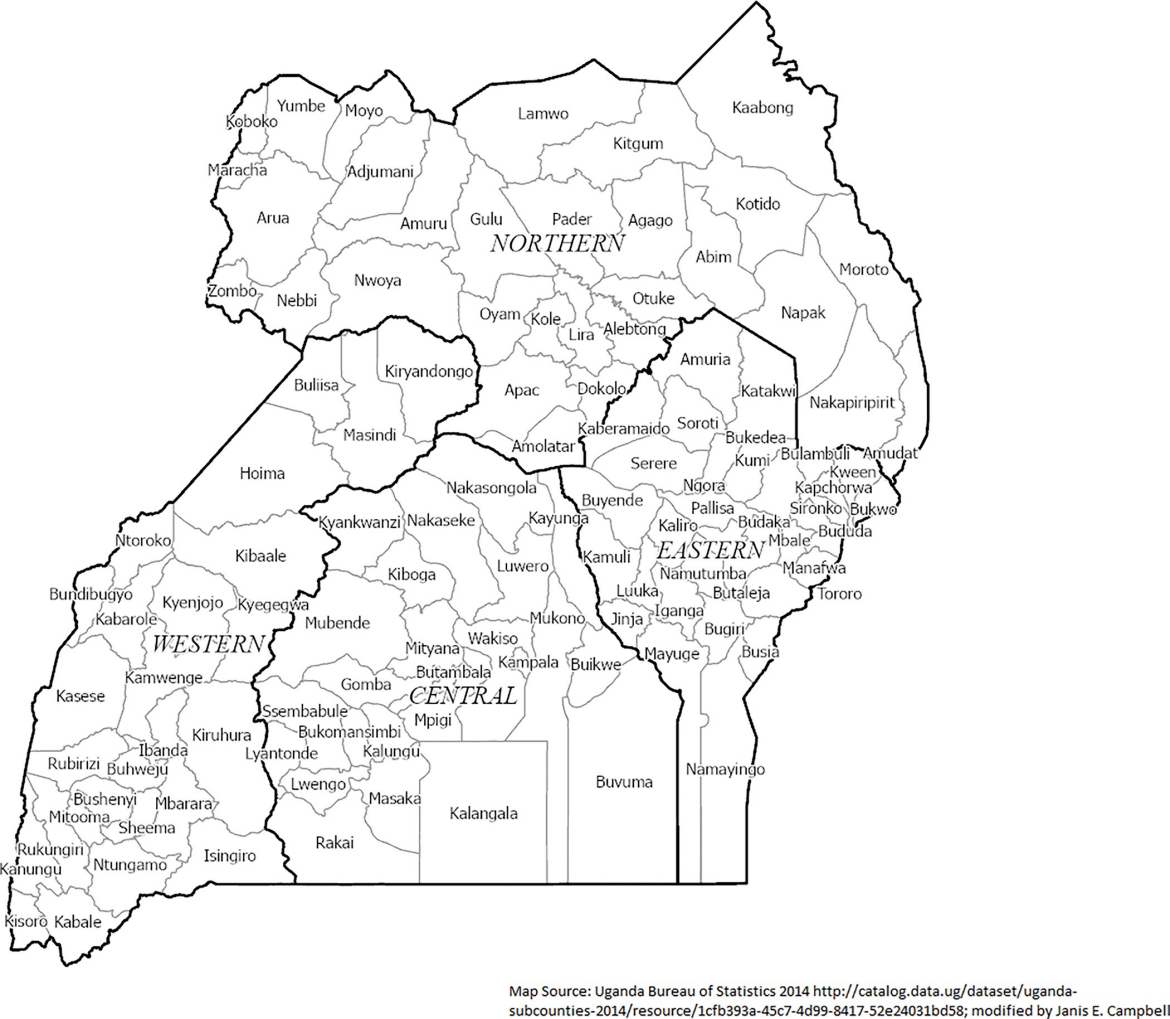
Study districts (n = 112) and regions (n = 4) from the Uganda 2016 Demographic and Health Survey.

The Republic of Uganda is in East Central Africa. Uganda is a country of 241,551 square kilometers bordered by South Sudan, Kenya, Tanzania, Rwanda, and the Demographic Republic of the Congo. Over forty languages are spoken in Uganda; Swahili and Luganda are popular languages with English being the language of government and commerce [19]. Kampala is the capital city, the largest city, and the commercial capital of Uganda and is in the southwestern region (**Fig 1**). Also, located within southern Uganda is the historic Kingdom of Buganda, the oldest and largest centralized monarchy in Africa. While they do not manage government services, the kingdom effects important cultural factors. Finally, Uganda is well known for their acceptance of refugees and had over 1.4 million refugees located mostly in resettlement camps throughout the country in 2020 [20].

The most recent estimates show a population of 41.6 million persons in the country. Three out of four Ugandan households live in rural areas [21]. Administrative districts in Uganda in 2019 consisted of four (4) regions, 15 sub-regions, 135 districts, 309 counties, one city (Kampala) council and thirteen municipalities [22]. For this study, nine Ugandan areas were included in the 2014 Census data but not in the Uganda Demographic and Health Survey (UDHS) district data in 2016 as there were112 total districts. Because of the important growth and development of Uganda, there are variations through time of the geographic location of districts in Uganda. **Table 1** lists how these inconsistent 2016 districts and were allocated to be consistent from the 2014 Uganda shapefile.

**Table 1:** Edits made by districts Uganda infant mortality study 2016.

District	Date Began	Before (2014)	Completed (2016)
Bunyangabu	July 1, 2017	Bunyangabu county in Kabarole District	Merged with Kabarole District
Butebo	July 1, 2017	Butebo County in Pallisa District	Merged with Pallisa District
Kagadi	July 1, 2016	Part of Kibaale District	Merged with Kibaale District
Kakumiro	July 1, 2016	Part of Kibaale District	Merged with Kibaale District
Kyotera	July 1, 2017	Part of the Rakai District	Merged with Rakai District
Namisindwa	July 1, 2017	East Bubulo County in Manafwa District	Merged with Manafwa District
Omoro	July 1, 2016	Omoro County in Gulu District	Merged with Gulu District
Pakwach	July 1, 2017	Part of the Nebbi District	Merged with Nebbi District
Rubanda	July 1, 2016	Rubanda County in Kabale District.	Merged with Kabale District.

### Data collection

For this project, two primary datasets will be used—the 2014 Uganda Bureau of Statistics (UBOS) census shapefile and the 2016 United States Agency for International Development (USAID) Demographic and Health Survey (DHS) for Uganda. Uganda’s Bureau of Statistics administers both the Ugandan Census (completed in 2014) and the Ugandan Demographic and Health Survey (UDHS) (completed in 2016). The DHS has created a new modelled estimates for small area estimation of health and demographics [23,24]. For this study, the unit of analysis was district, thus the DHS GIS files from DHS were not used.

The UDHS was sixth in a series of DHS conducted in Uganda in 1988–89, 1995, 2000–01, 2006, and 2011 (https://dhsprogram.com/data/). The main objective of the 2016 UDHS is to provide up-to-date information on fertility and childhood mortality levels; fertility preferences; awareness, approval, and use of family planning methods; maternal and child health; domestic violence; knowledge and attitudes toward HIV/AIDS; and maternal mortality. The information collected through the 2016 UDHS proposes to assist policymakers and program managers to design and evaluating programs and strategies intended to improve the health of Uganda. Design weights were adjusted for household nonresponse and individual nonresponse to obtain the sampling weights for households and for women and men, respectively.

### Dependent variable

Infant mortality is the death of an infant in the first year of life. They are categorized as yes (infant who died before their first birthday) and no (infant who lived past their first birthday). The dependent cases in this file include the children ever born of eligible women. There were 57,906 eligible women in the dataset.

### Independent variables

Socio-demographic factors included mothers age, mothers educational, language spoken in interview (English or Luganda), literacy, marital status, occupation of the mother, smoking, previous death of a baby, age at first birth, contraception use, distance to health care and intended pregnancy. Pregnancy factors included number of antenatal visits, during pregnancy, given or bought iron tablets/syrup, during pregnancy took: SP/fansidar for malaria. Infant factors included sex, birth order, and birth weight. Delivery factors included pre-term birth and birth interval. Geographic factors included urban and rural areas. Household factors included overcrowding, electricity, water sources, type of cooking fuel, wealth, household has radio, household has bicycle and household has television. Father’s factors included father’s education and occupation of father.

### Geographic and statistical analysis

Given the complex multifactorial nature of most infant deaths, to understand the health of a community, modeling multiple factors is necessary. When these relationships are strictly linear, we can use traditional statistics such as Ordinary Least Square (OLS) or logistic regression, however when the relationships have spatial heterogeneity, a more complex process is necessary to deal with place. In the first stage, exploratory OLS modelling was completed. The OLS provided coefficients averaged over the entire area. A test for spatial autocorrelation (Moran’s I) of the residuals was then completed to determine if the model data were or were not clustered. To control for residual autocorrelation and heteroscedasticity, a second stage, identified as necessary in the OLS estimation, used a local approach, semi-parametric and multi scale GWR [25,26]. GWR is widely used for fitting data displaying spatial non-stationarity [25,27–30]. For this study, we used the GWR in ESRI® ArcGIS Pro 3.0. We used a distance band neighborhood type with a Golden search selection method.

This study used UBOS collected DHS data for our dependent variable of infant deaths rates (number of infant death/number of births*100) at the district level. Tests for normality were performed using Proc Univariate in SAS 9.4. Additionally, exploratory analysis in ArcGIS was used to wean down to a significant exploratory analysis. Variables were initially chosen from a review of the literature then only significant variables from the OLS remained in the model. If a variable was determined to not be normally distributed a natural log was used. Each of the variables with 0 in the weighted frequency were changed to 0.00001 for analysis purposes. Significance at the 0.05 level was used.

### Ethics statement

The analysis was a secondary analysis conducted on publicly available data from Uganda. The study project did not involve any significant risk to study participants. The necessary data use agreements to obtain the Uganda specific data were completed and approved. This project was reviewed by the University of Oklahoma Health Sciences Center Institutional Review Board and determined not to be human subjects’ research. The project was also reviewed and approved by the University of Salford ethics review board.

## Results

### Overall results

**Fig 2:**
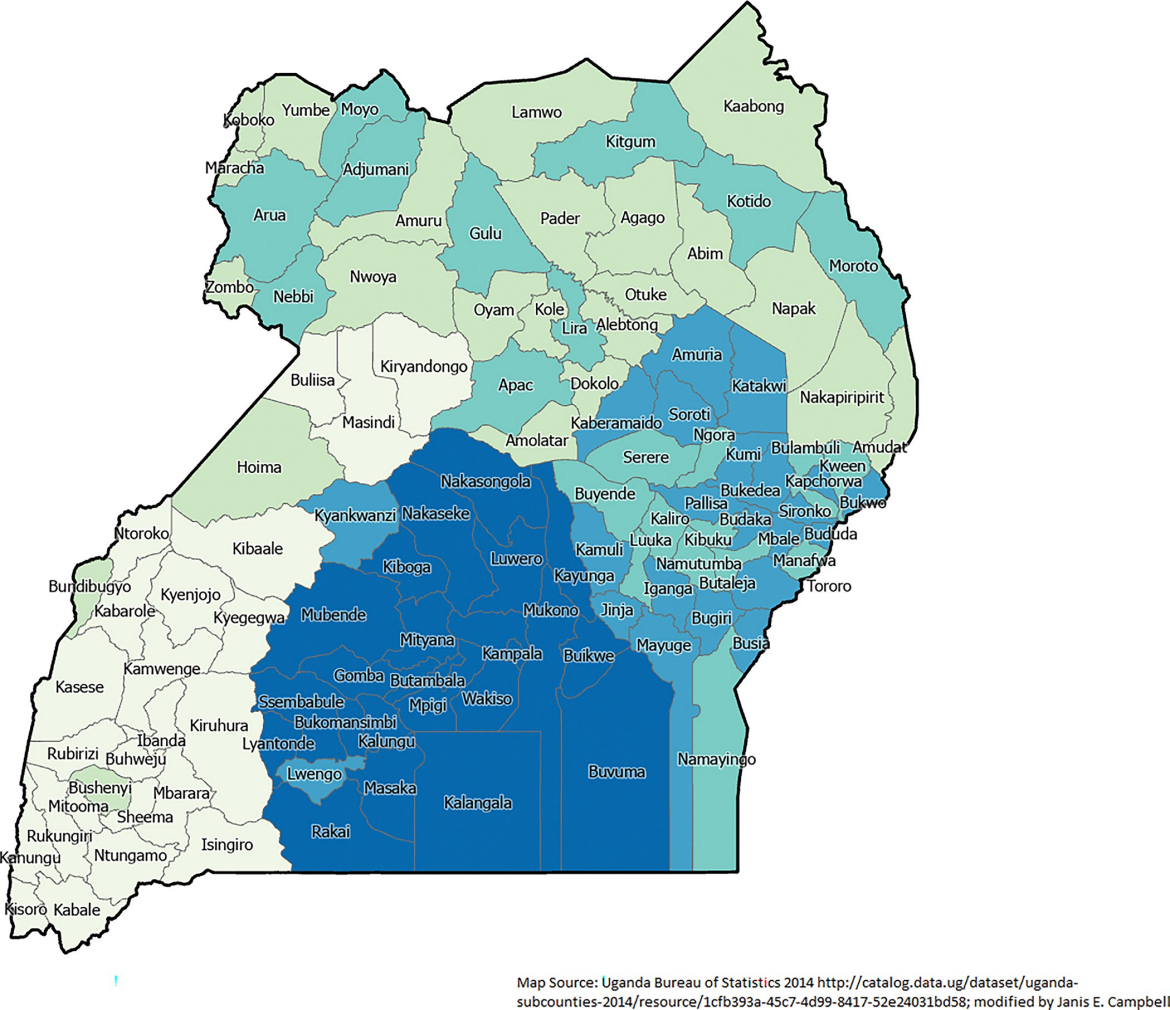
Infant mortality rate by district (n = 112) from Uganda 2016 Demographic and Health Survey.

An estimated 6.1% of births died the first year of life in 2016. The range of IM was from 2.8% of births dying in Shema District to 12.4% in Kalangala District (**Fig 2**). The total number of polygons in the area was 112. There appears to be a strong cluster of high IM in the south central region of Uganda. In the existing literature, 31 variables were readily available for analysis from the DHS survey. **Table 2** summarizes the significant Chi-square measure for each of these exploratory variables. Through exploratory analysis, only 14 variables were kept for the Ordinary Least Square analysis and the subsequent GWR analysis.

**Table 2:** Chi-square analysis of variables related to poor infant health and risk factors from Uganda 2016 Demographic and Health Survey: Final exploratory independent variables are highlighted and italicized.

Variable	IM
**Maternal Characteristics**	
Maternal Age	< .0001
Education	< .0001
*Language of Interview/Maternal Native*	*0*.*0076*
Literacy	< .0001
*Marital Status*	*0*.*0346*
Occupation Mother	0.0104
Smoking	< .0001
Previous death of a baby	< .0001
Age at first birth	0.0042
Contraception Use/Method	0.0256
Distance to health care	0.008
Intended pregnancy	0.0559
**Pregnancy**	
Antenatal visits	< .0001
During pregnancy, given or bought iron tablets/syrup	0.0002
During pregnancy took: SP/fansidar for malaria	0.0002
**Infant Characteristics**	
Sex	0.0003
Birth order	< .0001
Birth weight	< .0001
**Delivery Characteristics**	
Pre-term Birth	< .0001
Birth Interval	< .0001
**Geographic Characteristics**	
Urban/Rural	0.0077
**Household Characteristics**	
Overcrowding	0.0002
Electricity	0.0004
Water Sources	< .0001
Type of cooking fuel	< .0001
Wealth	0.0002
Household has: radio	0.0044
Household has: bicycle	0.0499
Household has: television	0.0001
**Father’s Characteristics**	
Father’s education	< .0001
Occupation Father	0.0408

### Spatial Autocorrelation

**Fig 3:**
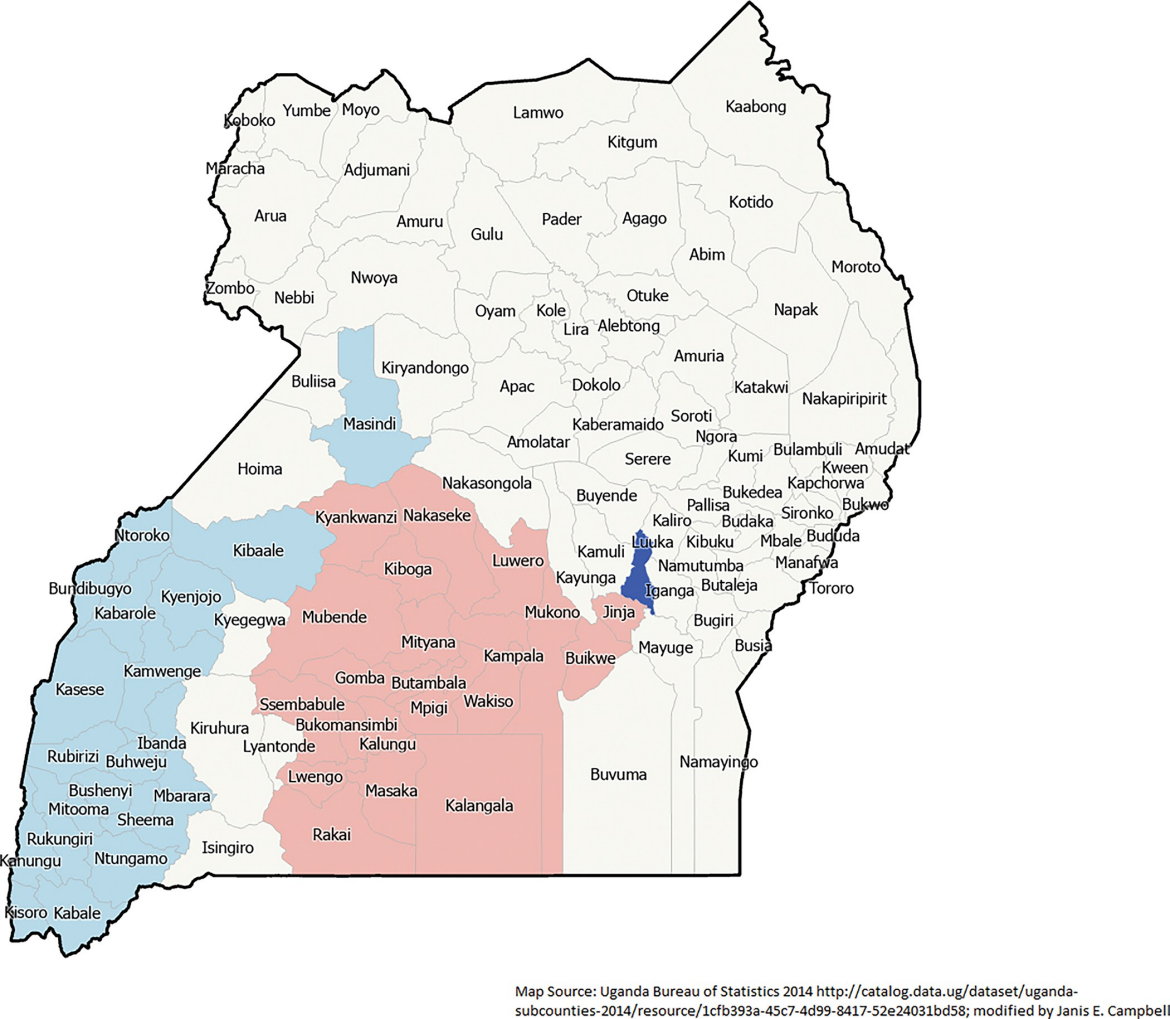
Infant mortality clusters in study districts (n = 112) using Anselin Local Moran’s I: from Uganda 2016 Demographic and Health Survey.

Spatial Autocorrelation (Global Moran’s I) is a way to determine the randomness of geographic events. Through a Global Moran’s I, we see that IM was clustered (p-value < .00001; z = 11.823). The local Moran’s I shows a clear cluster of high-high IM in the southcentral part of Uganda and a low-low cluster in southwest Uganda (**Fig 3**).

### Ordinary Least Square (OLS)

Using the OLS backward regression, the final models explained 33.8% of IM variance (AICc 426.105). Among infant deaths, having completed the interview in the language Luganda had the highest t-statistic (6.0938) and thus was the most important explanatory variables in the model (p-value < .0001). Being unmarried was significant (T-statistics 2.725; p-value 0.004) as well. Using the Anselin Moran’s I, IM was significant for spatial dependence or clusters (z-score 8.979015; p-value < .0001). The local Moran’s I shows several significant clusters (**Fig 3**); the model shows some residual low clustering in two areas of the eastern region, Nakapiripirit District in the northern region and Lwengo District in the central region. Because of spatial heterogeneity processes, as shown by local Moran’s I, it was crucial to move to the second stage of the analysis plan using GWR.

### Geographically weighted regression

**Fig 4:**
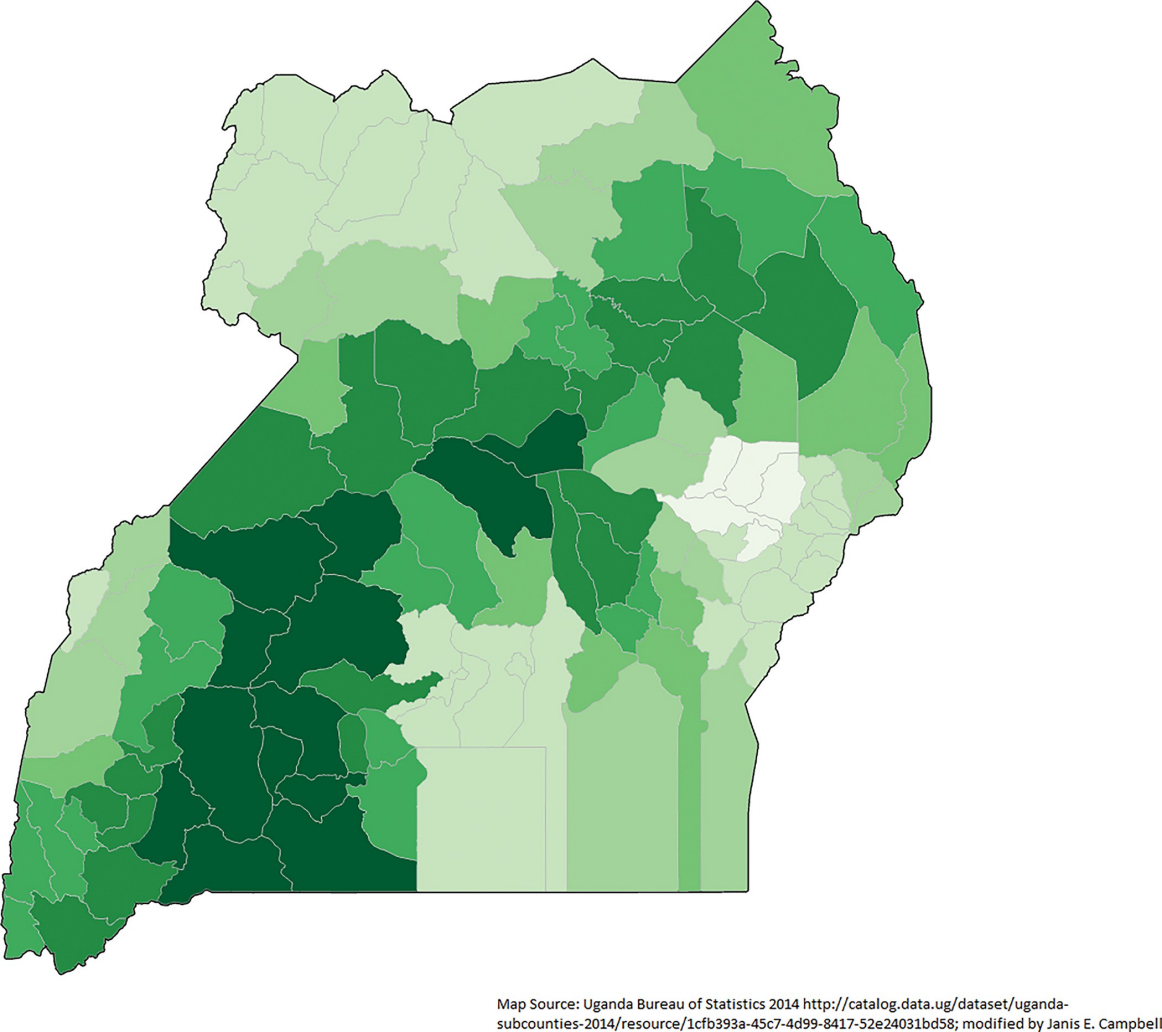
Geographically weighted regression of infant mortality by district (n = 135) for women giving birth in the last five years residuals controlling for marital status and being interviewed in Luganda Uganda 2016 Demographic and Health Survey.

Using GWR, the adjusted r-squared was 74.4% and the AICc was 340.1857, suggesting that this is a better method compared to the OLS global method. The adjusted r-squared for the IM full model ranged from only 0.002% to 83.2% for districts. The optimal bandwidth size from adaptive weighting scheme was 32 (out of 112 districts), which indicated very strong local effect or scaling effect. VIF values were far less than 7.5 meaning that there are not statistically significant multicollinearity issues. The residuals were not significant in spatial autocorrelation as show by a Global Moran’s I (p-value 0.4085; z-score 0.8265). Spatial dependence for districts by proportion of women giving births in the last five years and not being married showed more impact in central Uganda (**Fig 4**).

**Fig 5:**
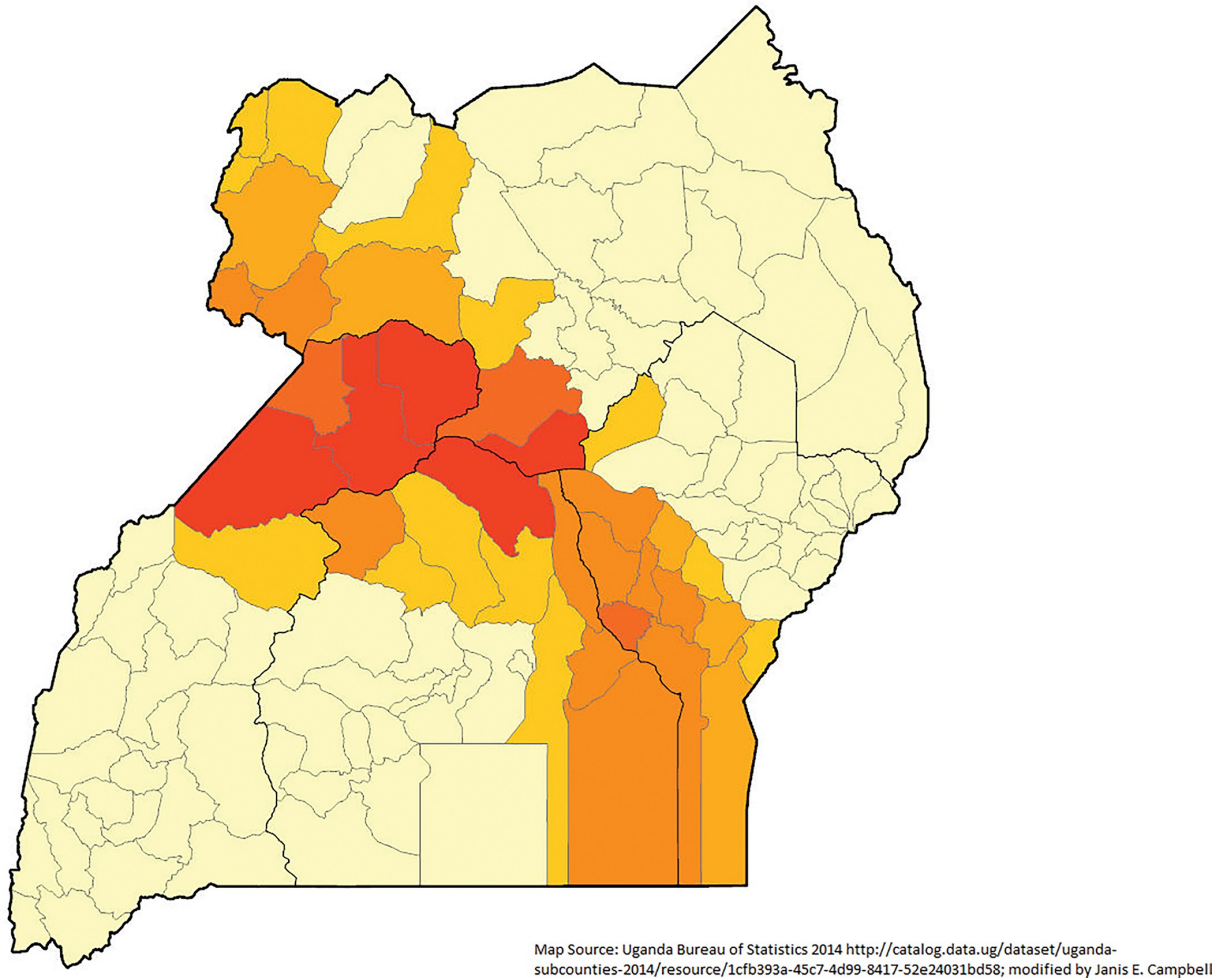
T-statistic estimates for unmarried women by district (n = 135) for women giving birth in the last five years and having an infant die in the first year of life Uganda 2016 Demographic and Health Survey.

**Fig 6:**
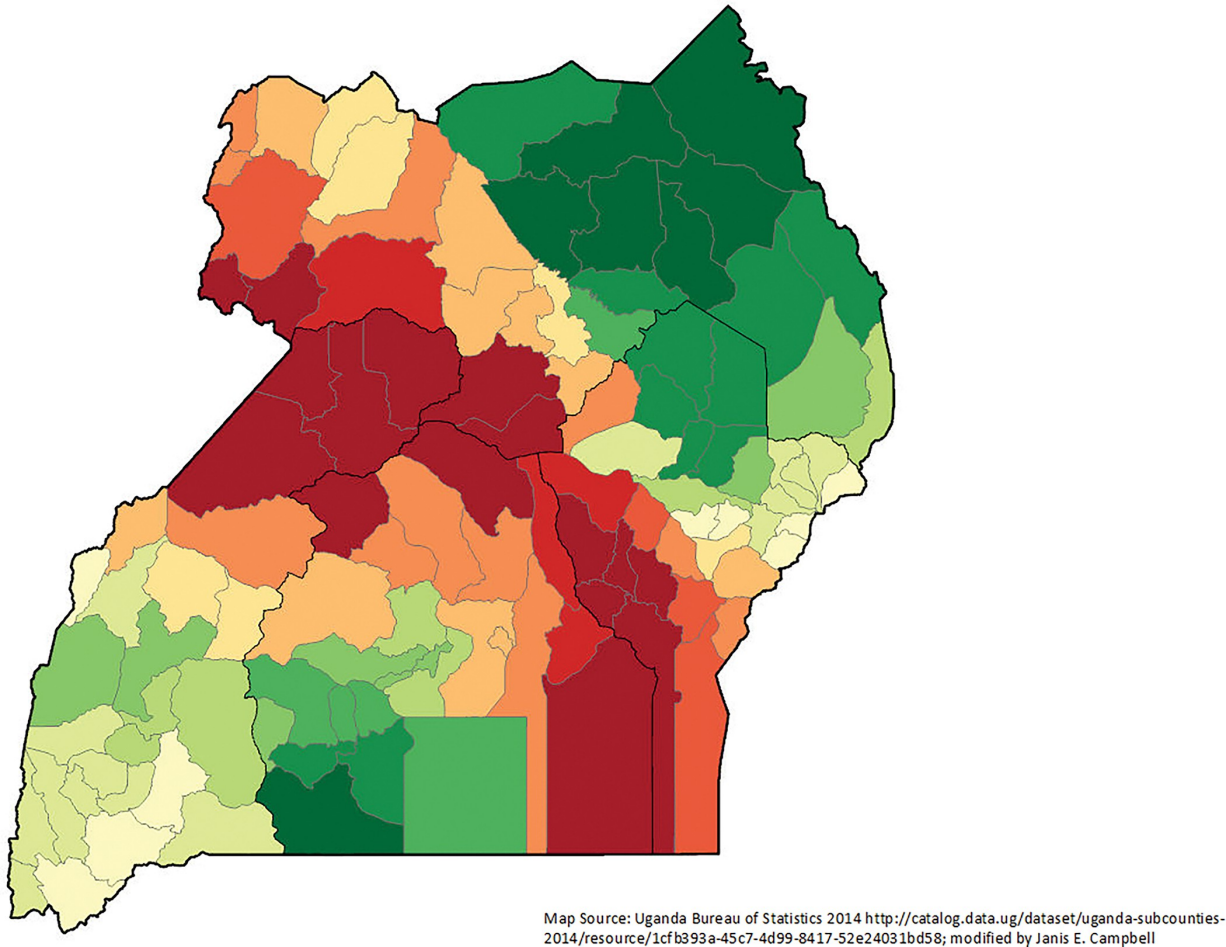
Parameter estimates for unmarried women by district (n = 135) for women giving birth in the last five years and having an infant die in the first year of life Uganda 2016 Demographic and Health Survey.

**Fig 7:**
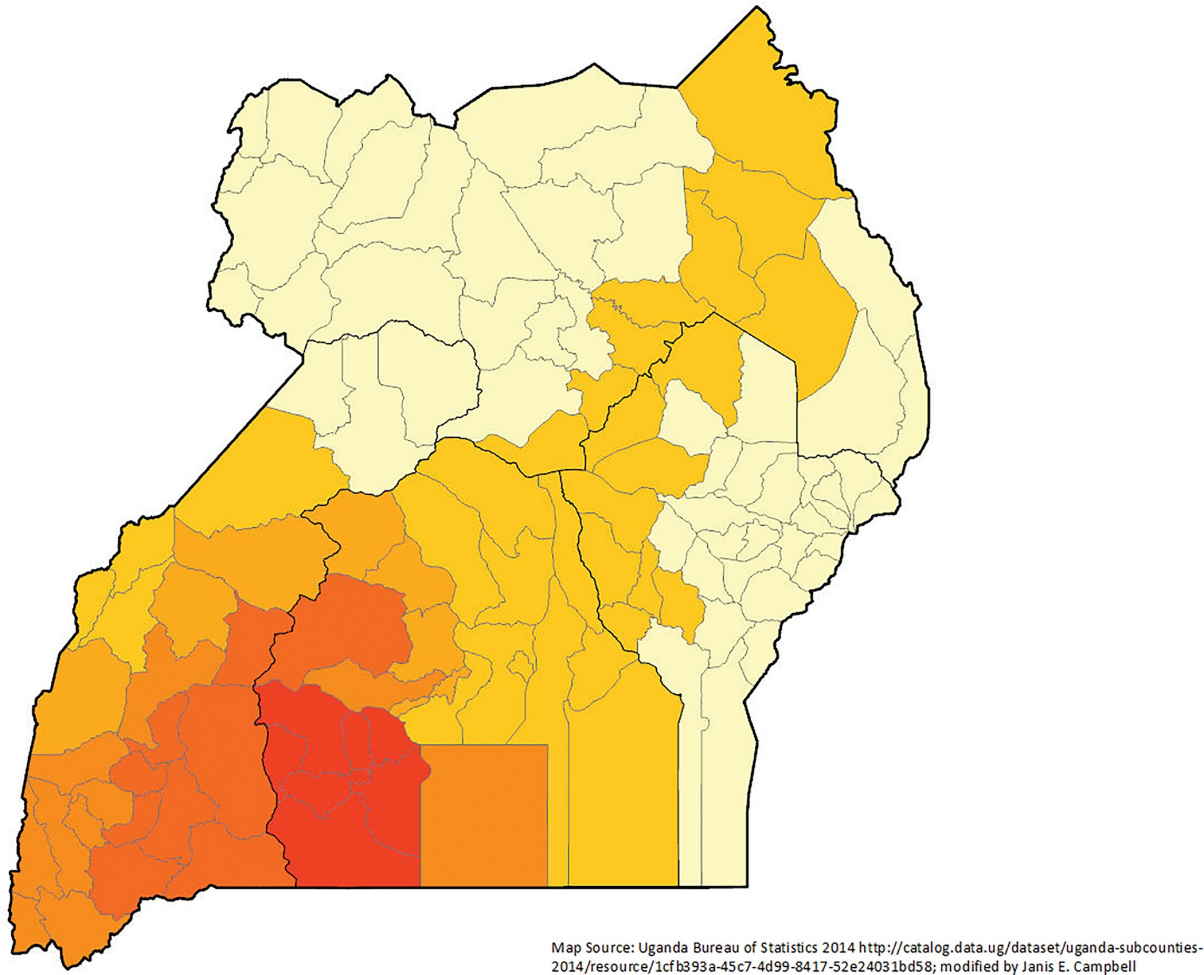
T-statistic for being interviewed in Luganda by district (n = 135) for women giving birth in the last five years and having an infant die in the first year of life Uganda 2016 Demographic and Health Survey.

**Fig 8:**
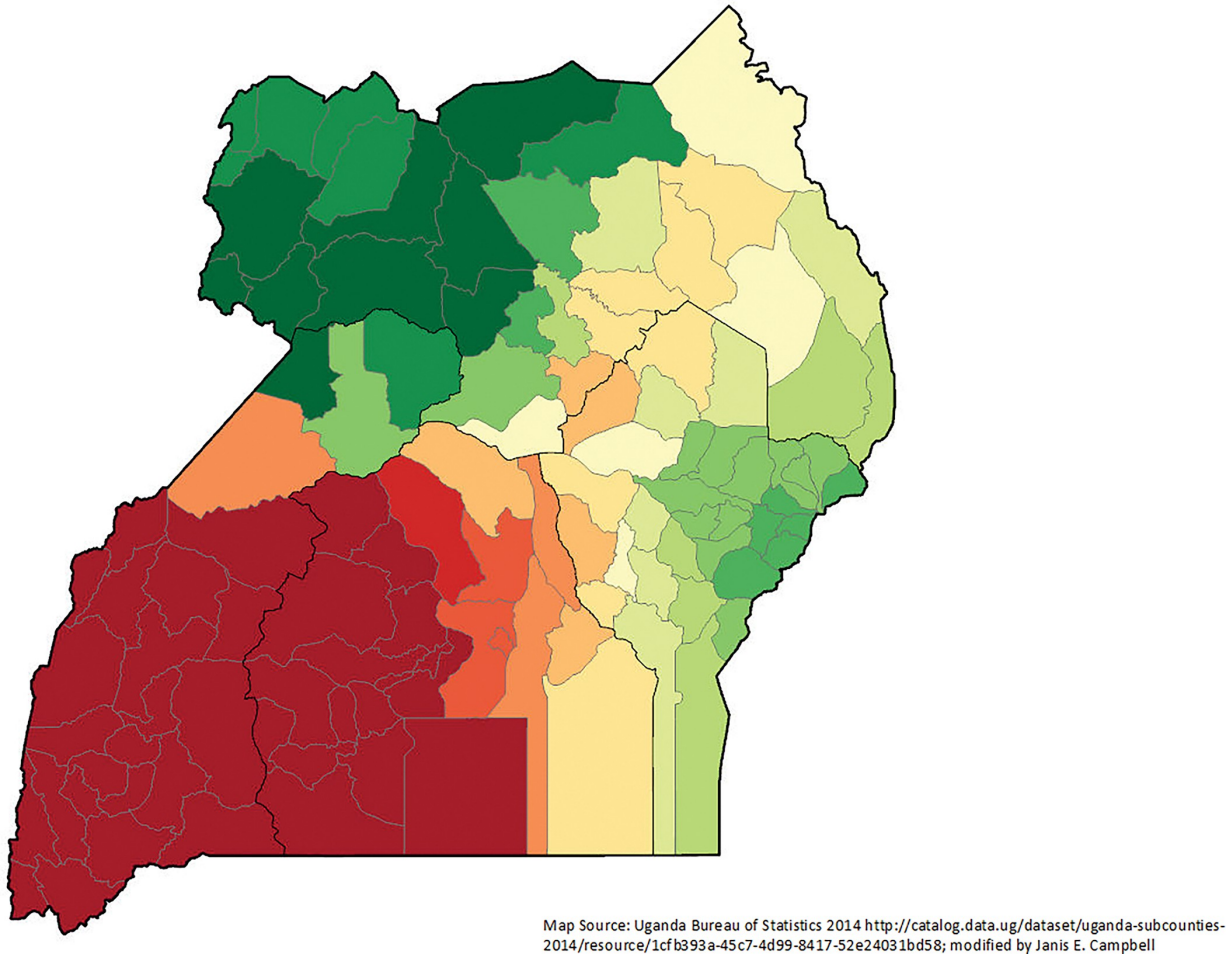
Parameter estimates for being interviewed in Luganda by district (n = 135 for women giving birth in the last five years and having an infant die in the first year of life Uganda 2016 Demographic and Health Survey.

Many high r^2^ districts (districts that were explained well by the model) were in the south-central part of Uganda (**Fig 4**). There appeared to be a geographic cluster of districts with women who were unmarried when they gave birth in the last five years significantly explaining IM in central Uganda (**Figs 5 and 6**). The local T-statistics ranged from -0.002 to 4.742; nine districts were significant at the 95% confidence interval (4.35 or higher). Among districts where the women interviewed preferred Luganda and had given birth in the last five years, there is a geographic cluster of high IM in south and southwestern Uganda (**Figs 7 and 8**). The local T-statistics ranged from -1.289 to 9.800; 42 districts were significant at the 95% confidence interval (4.35 or higher).

## Discussion

GWR allows for the development of local level estimates and compares their neighbors to produce maps and statistics that look at the processes behind spatial heterogeneity, while still comparing to a larger unit or the global process. One of GWR’s strengths is that it can be used in estimating parameters anywhere within the study area provided a dependent variable along with a set of one or more variables that have already been measured. This study showed the strengths of GWR by showing that districts with high rates of being unmarried and preferring to speak Luganda for their UDHS interview were significantly associated with infant deaths, but in different locations. These are both what we consider proxy measures or measures that represent something more than just those characteristics. As we know, being unmarried at the birth of a child often represents a lack of a social network to assist with income, childcare, and household chores, thus may represent an area with decreased resources consequently in higher need. Additionally, being interviewed in Luganda was associated in a large area, which may represent not being comfortable speaking English, which is the language of education, commerce, and presumably health care, thus suggesting a disconnect with health care settings. Luganda is spoken in the highly populated south- and south-central parts of Uganda and therefore is a common spoken language.

These data suggest that strides can be made in Uganda by providing targeted resources to areas with high rates of unmarried mothers and with high rates of Luganda as their language of choice. Moreover, these districts are focused specifically on areas of west-central and south-southwestern regions of Uganda. These data suggest as well that these are areas of high IM where strong local associations exist (with high local r^2^ values). In short, these variables were the only two that reached significance among all variables suggesting that they are important explanatory variables, though they are not causal but associated with IM.

This study is at risk for ecological fallacy and no assumption of an individual’s or even household differences should be made. In addition, the modifiable areal unit problem (MAUP) must be considered. While these are the smallest geographic units available, they are administrative units and not natural boundaries [31–33]. By using the adjusted bandwidth MAUP, the different size of units and edge issues are less problematic, but still need to be considered [34]. The uncertain geographic context problem, that individuals interact differently not only than each other but at different times and in different contexts, must also be considered [35,36]. We used the district of residence rather than the UDHS GIS files for geographic location thus we did not need to consider the masking of GPS locations [23]. The survey utilized a complex survey design, thus understanding the weighting and sample selection is important to understand outcomes and limitation.

Despite the varied strengths of GWR, there are some potential limitations. To begin with, database errors such as selective omission from birth histories among deceased infants which may be due to interviewer bias or even respondent fatigue or bias [37]. Secondly, the GWR works effectively only in the case where independent variables have a positive correlation. The method does not work correctly when independent variables negatively correlate [15]. Another drawback of GWR is that it assumes that every relationship being modeled diverges at one single scale; this consequently limits the ability to characterize spatial context. Similarly, with GWR, the same bandwidth is presumed to be applicable for every relationship within the model, which implies that data are weighted at a standard spatial scale. GWR is unable to alleviate the occurrence of an outlier and spatial autocorrelation that tends to pose a unique challenge resulting in instability in its spatial estimates. It is not clear how cluster sampling impacts this study. Lastly we used the weighted percentages for each district, thus, there should be limited bias due to this factor.

Despite these limitations, the findings of different associations in specific geographic areas are still important due to the high rates of IM in Uganda. Moreover, this article provides some high-risk areas of IM and their associated independent variables. The results invite further studies of social and demographic characteristics associated with IM throughout Uganda [23].
